# Optimization of Mesenchymal Stromal Cell (MSC) Manufacturing Processes for a Better Therapeutic Outcome

**DOI:** 10.3389/fimmu.2022.918565

**Published:** 2022-06-09

**Authors:** Maria Eugenia Fernández-Santos, Mariano Garcia-Arranz, Enrique J. Andreu, Ana Maria García-Hernández, Miriam López-Parra, Eva Villarón, Pilar Sepúlveda, Francisco Fernández-Avilés, Damian García-Olmo, Felipe Prosper, Fermin Sánchez-Guijo, Jose M. Moraleda, Agustin G. Zapata

**Affiliations:** ^1^ Cardiology Department, HGU Gregorio Marañón. GMP-ATMPs Production Unit, Instituto de Investigación Sanitaria Gregorio Marañón (IiSGM). Complutense University, CIBER Cardiovascular (CIBERCV), ISCIII, Madrid, Spain; ^2^ Platform GMP Units from TerCel and TERAV Networks. RETIC TerCel & RICORS TERAV, ISCIII, Madrid, Spain; ^3^ New Therapies Laboratory, Health Research Institute-Fundación Jiménez Díaz University Hospital (IIS-FJD). Surgery Department, Autonoma University of Madrid, Madrid, Spain; ^4^ Hematology Department and Cell Therapy Area, Clínica Universidad de Navarra. CIBEROC and IDISNA, Pamplona, Spain; ^5^ Hematopoietic Transplant and Cellular Therapy Unit, Instituto Murciano de Investigación Biosanitaria IMIB-Arrixaca, Virgen de la Arrixaca University Hospital, University of Murcia, Murcia, Spain; ^6^ Cell Therapy Area and Hematology Department, IBSAL-University Hospital of Salamanca, University of Salamanca, Salamanca, Spain; ^7^ Regenerative Medicine and Heart Transplantation Unit, Instituto de Investigación Sanitaria La Fe, Valencia, Spain; ^8^ Department of Cell Biology, Complutense University, Madrid, Spain

**Keywords:** MSCs, ATMPs, legal requirements, GMP manufacturing, extracellular vesicles

## Abstract

MSCs products as well as their derived extracellular vesicles, are currently being explored as advanced biologics in cell-based therapies with high expectations for their clinical use in the next few years. In recent years, various strategies designed for improving the therapeutic potential of mesenchymal stromal cells (MSCs), including pre-conditioning for enhanced cytokine production, improved cell homing and strengthening of immunomodulatory properties, have been developed but the manufacture and handling of these cells for their use as advanced therapy medicinal products (ATMPs) remains insufficiently studied, and available data are mainly related to non-industrial processes. In the present article, we will review this topic, analyzing current information on the specific regulations, the selection of living donors as well as MSCs from different sources (bone marrow, adipose tissue, umbilical cord, etc.), in-process quality controls for ensuring cell efficiency and safety during all stages of the manual and automatic (bioreactors) manufacturing process, including cryopreservation, the use of cell banks, handling medicines, transport systems of ATMPs, among other related aspects, according to European and US legislation. Our aim is to provide a guide for a better, homogeneous manufacturing of therapeutic cellular products with special reference to MSCs.

## Introduction

Mesenchymal stromal cells (MSCs) are among the cell types most frequently used as therapeutic agents. Despite diverse approaches for improving their clinical efficiency, this remains low and is restricted to few diseases, including skeletal disorders, graft-versus-host disease and intestinal inflammation (1). Remarkably, protocols devoted to the clinical applications of MSCs are extremely variable, exhibiting differences in cell sources, banking processes, cell preservation, ways of administration, among others, and producing heterogeneous functionality of MSC products. In the present article, we review these issues, which have been significantly less investigated than the biology of MSCs used as therapeutic tools but, undoubtedly, important for the success of clinical trials. We also address the rules and legislations that govern these products of cell therapy. All steps from potential donor selection and manufacturing to cell transportation and administration to patients are reviewed. A section is devoted to MSC-derived extracellular vesicles (ECV) that are becoming an interesting therapeutic product whose generation, maintenance and administration have specific challenges. Our goal is to provide a general guide for a better and more homogeneous manufacturing of MSCs for use in cell therapy.

## Rules and Legislation for the Use of MSCs as Advanced Therapy Medicinal Products

Although cell therapy products have been produced for years for the treatment of different diseases, only in the first decade of the twenty-first century has the process of legally regulating their production and therapeutic use as medicines begun. In both the European Union (EU) and the United States (US), specific legislation has been established to approve the commercialization of cell and gene therapy products to ensure their quality, safety and efficacy (**Table 1**). The possibility of these products becoming medicines was initially addressed in the European Union (EU) by the first European directives on medicines (Directive 2003/63/EC and Regulation 726/2004/EC), but it was not until 2007 when a specific regulatory framework for so-called Advanced Therapies Medicinal Products (ATMPs) was introduced (Regulation 1394/2007/EC). Subsequently, the scientific and technical requirements for these ATMPs have been supplemented with successive directives (Directive 2009/120/EC, EU GMP-ATMPs).

**Table 1:** European Union and United States Legislation related with ATMPs.

EUROPEAN UNION LEGISLATION		
ABBREVIATION	LEGISLATION	DESCRIPTION
Directive 2003/63/EC	Commission Directive 2003/63/EC of 25 June 2003 amending Directive 2001/83/EC of the European Parliament and of the Council on the Community code relating to medicinal products for human use.	First Regulation on gene and cell therapy as medicines
Directive 2004/23/EC	Commission Directive 2004/23/EC of the European Parliament and of the Council of 31 March 2004 on setting standards of quality and safety for the donation, procurement, testing, processing, preservation, storage and distribution of human tissues and cells.	Regulation on donors and cells and tissues as starting materials
Directive 2015/566/EU	Commission Directive (EU) 2015/566 of 8 April 2015 implementing Directive 2004/23/EC as regards the procedures for verifying the equivalent standards of quality and safety of imported tissues and cells.	Selection leaving donors
2006/17/EC	Commission Directive 2006/17/EC of 8 February 2006 implementing Directive 2004/23/EC of the European Parliament and of the Council as regards certain technical requirements for the donation, procurement and testing of human tissues and cells.	Regulation on donors and cells and tissues as starting materials
Directive 2009/120/EC	Commission Directive 2009/120/EC of 14 September 2009 amending Directive 2001/83/EC of the European Parliament and of the Council on the Community code relating to medicinal products for human use as regards advanced therapy medicinal products.	Regulation on the scientific and technical requirements of ATMPs
Regulation (EC) No 726/2004	Regulation (EC) No 726/2004 of the European Parliament and of the Council of 31 March 2004 laying down Community procedures for the authorisation and supervision of medicinal products for human and veterinary use and establishing a European Medicines Agency.	First mention of ATMP as medicines
Regulation (EC) No 1394/2007	Regulation (EC) No 1394/2007 of the European Parliament and of the Council of 13 November 2007 on advanced therapy medicinal products.	Developed Regulation on ATMP
Regulation (EU) 2017/745	Regulation (EU) 2017/745 of the European Parliament and of the Council of 5 April 2017 on medical devices, amending Directive 2001/83/EC, Regulation (EC) No 178/2002 and Regulation (EC) No 1223/2009 and repealing Council Directives 90/385/EEC and 93/42/EEC.	Regulation on the combined use of Cell Therapy Products and Medical Devices
Regulation (EU) 2017/746	Regulation (EU) 2017/746 of the European Parliament and of the Council of 5 April 2017 on *in vitro* diagnostic medical devices and repealing Directive 98/79/EC and Commission Decision 2010/227/EU (Text with EEA relevance)	
**UNITED STATES LEGISLATION**		
21 CFR 1271	Code of Federal Regulations. Title 21 - Food and Drugs. Chapter I - Food and Drug administration. Department of Health and Human Services. Subchapter l - Regulations under certain other acts administered by the Food and Drug Administration. Part 1271 - Human cells, tissues, and cellular and tissue-based products.	Regulation on Human Cells, Tissues, and Cellular and Tissue-Based Products
21 CFR 211	Code of Federal Regulations. Title 21- Food and Drugs. Chapter I - Food and Drug Administration Department of Health and human services. Subchapter C - Drugs: General. Part 211: Current Good Manufacturing Practice for finished pharmaceuticals.	Current Good Manufacturing Practices (cGMP)
21 CFR 312	Code of Federal Regulations. Title 21- Food and Drugs. Chapter I - Food and Drug Administration Department of Health and human services. Subchapter D -- Drugs for human use. Part 312: Investigational New Drug application.	Investigational New Drug Requirements
21 CFR 600.	Code of Federal Regulations. Title 21- Food and Drugs. Chapter I - Food and Drug Administration Department of Health and human services. Subchapter F - Biologics. Part 600: Biological products: General	Biologics License Application Requirements
42 USC 262.	United States Code. Title 42 - The Public Health and Welfare. Chapter 6A - Public Health Service. Subchapter II - General powers and duties. Part F - Licensing of Biological Products and Clinical Laboratories. Subpart 1 - biological products. Sec. 262 - Regulation of biological products.	Regulation of biological products

Gene Therapy products, Somatic Cell Therapy products, Tissue Engineering products and their combinations are considered ATMPs if they contain genes, cells or tissues that have undergone substantial manipulation (Regulation 1394/2007/EC, Annex I) that affects biological characteristics, physiological functions, or structural properties relevant for their clinical use. They also include cells or tissues that are used for different functions than their original ones, or in different locations in the recipient than in the donor. It is important to remark that products of cell therapy are considered as different from tissues or organs used for transplantation at a regulatory level, in that cell therapy products are considered to be medicines (ATMPs). Cell therapy products are also regulated by the guidelines of medical devices, Regulation (EU) 2017/745 and Regulation (EU) 2017/746 when these are used in combination with medical devices (**Table 1**).

MSCs meet the requirements to be ATMPs. They undergo substantial manipulations such as cell culturing or, sometimes, chemical (i.e. Fucosylation) or gene modifications (1). Moreover, they are obtained from different sources and can be used for a wide variety of applications. Besides, the European Medicine Agency (EMA) responsible for evaluating marketing commercialization of ATMPs through the Committee on Advanced Therapies-CAT (2) considers these products to be special medicines and their production to follow its own quality standards (see the Guidelines on Good Manufacturing Practice specific to Advanced Therapy Medicinal Products - EU ATMPs-GMP **Table 2**).

**Table 2:** Guidelines, ISOs (International Organization for Standardization) and rules related with ATMPs.

GUIDELINES and RULES			
ABBREVIATION	TITLE	DESCRIPTION	APPLY TO
EU GMP-ATMP	EudraLex-The Rules Governing Medicinal Products in the European Union. Volume 4: Good Manufacturing Practice. Guidelines on Good Manufacturing Practice specific to Advanced Therapy Medicinal Products. 22 November 2017.	Good Manufacturing Practice specific ATMPs	EU
CMCa	Guidance for FDA Reviewers and Sponsors: Content and Review of Chemistry, Manufacturing, and Control (CMC) Information for Human Gene Therapy Investigational New Drug Applications (INDs) (2008).	FDA guidance on Chemistry, Manufacturing, and Control of Gene Therapy products	US
CMCb.	Guidance for FDA Reviewers and Sponsors: Content and Review of Chemistry, Manufacturing, and Control (CMC) Information for Human Somatic Cell Therapy Investigational New Drug Applications (INDs) (2008).	FDA guidance on Chemistry, Manufacturing, and Control of Somatic Cell Therapy products	US
(WHO) EB123/5	Executive Board, 123. (2008). Human organ and tissue transplantation: report by the Secretariat. World Health Organization. https://apps.who.int/iris/handle/10665/23650.	WHO guiding principles on human cell, tissue and organ transplantation	BOTH
WHA57.18	Resolution of 2009: Human organ and tissue transplantation (https://apps.who.int/gb/ebwha/pdf_files/WHA57/A57_R18-en.pdf)	Resolution on organ procurement and Allogenic/Xenogeneic transplantation	BOTH
EMEA/CHMP/410869/ 2006	Guideline on human cell-based medicinal products	Development, manufacturing and quality control, and non-clinical and clinical development of cell-based medicinal products. It covers somatic cell therapy medicinal products and tissue engineered products.	EU
FDA-2008-D-0520	Guidance for Industry: Potency Tests for Cellular and Gene Therapy Products (01/2011)	Recommendations for Potency Assay design in cellular and gene therapy products.	US
ICHQ5D	Quality of Biotechnological Products: Derivation and Characterization of Cell Substrates Used for Production of Biotechnological/Biological Products. CPMP/ICH/294/95. 1998.	Standards for the derivation of human and animal cell lines and microbial cells to be used in biotechnological/biological products	BOTH
CPMP/ICH/138/95	Note for guidance on quality of biotechnological products: stability testing of biotechnological/biological products	Generation and submission of stability data for well-characterized different products.	BOTH
CPMP/ICH/365/96	Note for guidance on Specifications: test procedures and acceptance criteria for biotechnological/biological products (ICHQ6B)	International specifications for biotechnological and biological products to support new marketing applications	BOTH
EMA/CHMP/BWP/534898/2008 (Rev. 2)	Guideline on the requirements for quality documentation concerning biological investigational medicinal products in clinical trials (27 January 2022)	Quality requirements of an investigational medicinal product for a clinical trial	US
GDP	Good distribution practice (https://www.ema.europa.eu/en/human-regulatory/post-authorisation/compliance/good-distribution-practice)	The minimum standards that a wholesale distributor must meet to ensure that the quality and integrity of medicines is maintained throughout the supply chain	EU
ISO 21973	Biotechnology-General requirements for transportation of cells for therapeutic use. (https://www.iso.org/obp/ui/es/#iso:std:iso:21973:ed-1:v1:en)	General requirements and reviews the points to consider for the transportation of cells for therapeutic use, including storage during transportation.	BOTH

US regulations also classify gene therapy and cell therapy products as biological products (42 USC 262), distinguishing them from conventional drugs. Traditional transplantation of cell or tissue products (Human Cell, Tissue and Cellular and Tissue-based product - HCT/P) is also different from that of biologicals (21 CFR 1271). As in the European legislation, HCT/P are characterized by their minimal manipulation and homologous use. Besides, they cannot produce systemic effects and their potential effects do not depend on the metabolic activity of living cells (3, 4). HCT/P intended for non-homologous use, or substantially modified, are regulated as biological products and will be included within the regulations for new investigational drugs (21 CFR 312), biologics (21 CFR 600) and cGMP (21 CFR 211). In the US, all these products are regulated by the Center of Biologics Evaluation and Research (CBER) within the Food and Drug Administration (FDA) (5).

### Legal Requirements for Donor Selection

Both American and European legislation requires an adequate selection of the donor, a guarantee of the traceability of the donated cells and tissues, and their processing under quality conditions that ensure their safety. According to European Directive 2004/23/EC, the donations must be voluntary, and the donors should have appropriate information about the obtaining procedure and the future use of their donated cells or tissues. The confidentiality of donated cells and tissues must also be assured. Donor evaluation and testing procedures must be documented, and any major anomalies reported. Selection criteria are described in section 2. Procedures for donor selection are similar in the US (see American 21 CFR 1271).

In Europe, the authorization of Tissue establishments is granted according to the provisions of Directive 2004/23/EC of 31 March 2004 on setting standards of quality and safety for the donation, procurement, testing, processing, preservation, storage and distribution of human tissues and cells. These authorizations are usually specific to each type of tissue or cell obtained and are valid for a specified period of time, at the end of which they can be renewed upon verification that the conditions and requirements that gave rise to their concession persist. When the collection of tissues and/or cells have to be obtained in a non-authorized health center, the procedure must always be carried out by professionals integrated in a collection team from a properly authorized center and under the conditions set by this center. The collection team must also have the proper authorization for this specific practice.

The obtained tissues must be packed and labeled according to Directive 2004/23/EC and 2006/17/EC and delivery to the manufacturing centers must be done with temperature traceability and by a qualified transportation company (6).

### GMP Manufacturing

ATMPs manufacturing is very similar to conventional sterile medicines production with some particularities. In fact, both in the EU and in the US, this is conducted in accordance with the

Good Manufacturing Practice of Medicines (EU GMP-ATMPs and 21 CFR 211, respectively) (**Tables 1**, **2**).

The EU Part IV of Volume 4 of the Good Manufacturing Practice (see EU GMP-ATMPs guide) includes the guidelines that develop GMP requirements in accordance with EU Regulation 1394/2007/EC and Directive 2009/120/EC. Essentially, the protocol for obtaining starting materials (Bone Marrow, Adipose Tissue, Umbilical Cord, etc.) must be well-defined, materials used for collection and shipment must be controlled, and the shipment protocol must be validated to guarantee stability (at least composition, viability and microbiological safety). Complementary legislation would be applied to the manufacturing of ATMPs that have been granted a marketing authorization and ATMPs used in a clinical trial setting. In the US, the FDA has provided two guidance documents of regulations for the Chemistry, Manufacturing and Controls (CMC) for gene (see CMCa) and cell therapy (see CMCb) products under the term of new drug procedure (**Table 2**) (3). Therefore, the EU and US regulations reflect the differences between GMP that apply to conventional medicines and those that apply to ATMPs (2). The GMP-specific regulation for ATMPs summarizes all the main issues of nonconventional drug manufacturing supported on the risk-based approach. ATMPs-specific GMPs highlight the personnel qualification, as well as the qualification and validation of facilities, equipment, documentation, starting and raw materials and excipients, aseptic production, test methods and quality control, batch release and distribution.

### The Impact of the ATMPs Regulatory Framework on the Development of MSC-Based Therapies

The development of ATMPs has traditionally been associated with GMP facilities. On the one hand, they must comply with GMP to ensure the safety, quality and efficacy of the ATMPs produced, but there may be impediments in the EU to the implementation of all the requirements. For instance, it is necessary to provide pre-clinical data on the proposed medicine product and a qualified person (QP) for formal release of the ATMPs. In addition, the lack of standard procedures for the application of EU directives among EU member states makes it difficult to regulate certain cellular products (7).

Although regulations are similar, some aspects of US legislation make it easier to conduct the early stages of ATMPs development there. Unlike the EU, US GMP facilities for manufacturing phase I/II and phase II trials are not subjected to regulatory inspection, so the burden of compliance is lower. In the US, there is no requirement for QP the formal release of investigational medicines (8). On the other hand, the lack of advanced phase III trials explains why only a few MSC-based cell therapy products have been approved today for market commercialization world-wide. The first products approved corresponded to Queencell (autologous adipose tissue-derived MSCs (AT-MSCs) for subcutaneous tissue defect, 2010), HeartiCell gram (autologous bone marrow-derived MSC (BM-MSC) for myocardial infarction, 2011), Cartistem (allogenic umbilical-cord blood (UC-MSC) derived MSC for osteoarthritis, 2012) or Prochymal (allogenic BM-MSC for acute graft vs host disease, 2012), but nowadays all of them remain in the market. Since then, as of 2021, only ten MSC-based products have been approved worldwide (9). However, in the EU only one product has been developed (Alofisel, allogenic adipose tissue derived MSCs (ADSCs) for perianal fistula) and, to date, there is no FDA-approved MSC therapy on the market^1^. This situation is particularly evident in EU academic institutions, which have limited experience in the regulatory protocols. Therefore, to develop guidelines, interactive initiatives or platforms, some previously mentioned, would be particularly useful. In the EU, EMA offers personalized scientific advice about any stage of MSC product development (10).

In the EU, MSC-based products are also authorized under the hospital exception clause. Centralized marketing authorization is not required in the EU if the ATMPs are prepared on a non-routine basis, according to GMP, in a specific hospital under responsibility of a medical specialist to cover an individual medical prescription for a custom-made product for an individual patient (7).

## Selection of Living Donors

Manufacturing of cells for clinical applications begins with an accurate selection of living donors according to the legal/ethical rules. This selection includes both the tissue of origin and the donor person. Regarding the donor tissue, much has been written emphasizing that MSCs from different origins (adipose tissue, bone marrow, Wharton’s jelly, etc.) have some specific properties. However, little is known about the influence of the donor on the capabilities of MSCs. Accordingly, here we briefly summarize some minimal requirements for MSC donation. Before addressing this point, it seems interesting to board a crucial question: Are autologous or allogeneic MSCs the best for therapeutic application? In fact, autologous MSCs would potentially be the best product because immunological rejection is avoided, but they do have a high production cost, requiring two procedures for the patient: one for obtaining the cell product and a second for the cell implantation, and the time of availability of the cellular product is also increased. Allogeneic MSCs from selected donors have three fundamental advantages and have become the most frequently used MSCs for cellular treatments: they have lower production costs, provide shorter treatment times and, most importantly, are barely immunogenic, evading the host immune system (Immunoevasive) (11–13), although data in this respect are controversial (1). In terms of safety, allogenic MSCs are considered to have the same properties as autologous ones. Regarding the effectiveness, to our knowledge, the ALOFISEL trial was the first Phase III clinical assay performed with allogeneic ADSCs with significant efficacy (14, 15). On the other hand, the use of allogeneic cells allows the generation of cell banks derived from optimal donors, i.e. those that have MSCs with the highest anti-inflammatory and immunosuppressive potential.

In this respect, a fundamental requirement in an optimal donor would be the absence of pathogens, for which the following must be ruled out: HIV, HBV, HCV, *Treponema pallidum*, Toxoplasmosis, Parvovirus, Epstein-Barr virus, Cytomegalovirus, Nile Virus and prions, and donors must have two negative PCRs and a negative IgM antibody test for COVID-19. They would also be required to have normal routine test results (hematology, biochemistry), and an absence of the following: fever, signs or symptoms of concurrent bacterial, fungal or viral infections, neoplastic antecedents, blood transfusions and tattoos or piercing during 4-6 months. Finally, it is advisable that they have not travelled to areas at risk of infectious diseases in the previous three months (Official WHO Guiding Principles (EB123/5) and resolution WHA57.18 of 2009, **Table 2**).

Individuals who meet the requirements set out above can be MSC donors but, how to select those whose MSCs maintain their regenerative/reparative properties intact? In the case of ADSCs, some studies have been conducted to answer this question in relation to gender, age/microsatellite length, lifestyle habits (Tobacco/Alcohol, Sport), type of fat (white or brown) and body mass index (BMI) (16).

This research found that women yielded a higher number of ADSCs with better immunomodulatory potential (17). Also, distinct anatomical sites provided different MSC yields, with variations in their immunomodulatory and differentiation potential (18, 19). Studies evaluating senescence showed a significant decrease in the overall cellular yield with increasing age and, more importantly, a significant fall in the proliferation and differentiation capacities of the obtained MSCs (20–25). On the other hand, numerous reports have related lifestyle habits with MSC “quality”: various by-products of tobacco inhalation/consumption, especially nicotine, have a detrimental effect on the number and capacities of MSCs (26–29). It has also been shown that regular alcohol consumption leads to a lower potential of MSCs as well as to decreased MSC numbers, especially those originated in the bone marrow (30–32). In the case of ADSCs, it is unclear whether subcutaneous fat and omentum fat have similar capacities, although the initial yield at isolation is higher in the omentum per gram (33, 34); and finally, different studies have shown that the highest cell yield is obtained from donors with a BMI between 17.5 and 26.8 (16, 20, 35–41). In summary, the “ideal” donor to obtain ADSCs is a young woman (<40 years) with healthy lifestyle habits (no tobacco, alcohol or drugs), no excessive fibrous tissue (such as athletes), and a BMI lower than 26.8.

In the case of BM-MSCs, any person in good health and aged between 18 and 40 years may be a good candidate (Directive 2015/566/EU). Nevertheless, some studies do not recommend donors suffering from uncontrolled high blood pressure, insulin-dependent diabetes mellitus, any severe cardiovascular, neurological, pulmonary, renal, hepatic disease, etc. Other risk factors include intravenous drug abuse, sexual risk practices, hemophilia, etc.; history of ocular inflammatory diseases (iritis, episcleritis) or fibromyalgia, donors receiving lithium treatment platelet counts below 120,000 ml. or those weighing less than 50 kg or more than 130 kg (42–44).

With regards to the donations of UC-MSC, the requirements established by world legislation for the donors are: the mother’s clinical history particularly in relation to possible infectious, hematological or any other type of illnesses that might contraindicate the use of cord blood; analysis of the mother’s blood at the time of birth to rule out any infectious process that could be transmissible to the cord blood; and clinical examination of the baby at birth and advisable 3 months after the sample collection. To our knowledge there are no studies that have evaluated the best umbilical cord donor, either in relation to the age of the mother or race. Therefore, with the exception of safety data, it is not possible to propose criteria for selecting donors for this type of MSCs.

## Isolation and Expansion of MSCs Derived From Different Sources

MSCs have been isolated from numerous adult and perinatal tissues, more frequently adipose tissue, bone marrow and umbilical cord, but also from dental pulp, menstrual blood, amniotic fluid or others (45). Regardless of their origin, all these MSCs can satisfy the minimal criteria of the International Society for Cellular Therapy (ISCT) in terms of phenotype, differentiation and immunoregulatory capabilities (46), but the cell yield, growth kinetics and potency may be affected by the tissue of origin or the protocol followed to obtain the starting material for cultures (47–49). So, with the available knowledge to date, the selection of the starting material as well as the methods of cell isolation and expansion are based on a mixture of logistical, intellectual and center experience or industry arguments.

**Figure 1:**
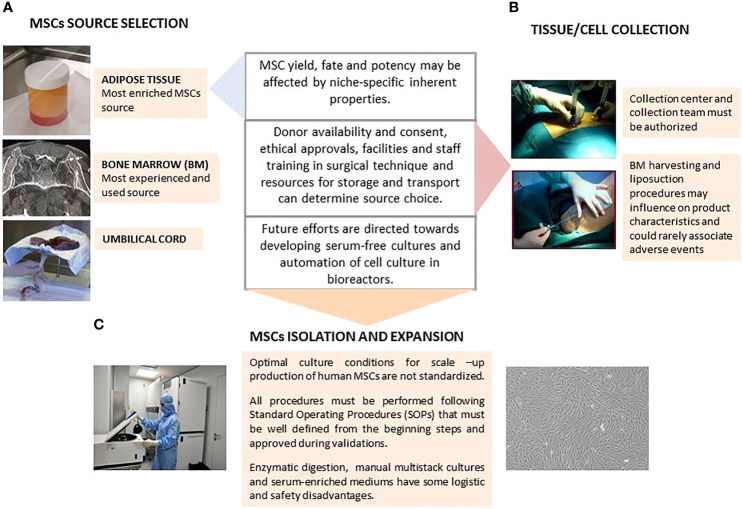
Schematic representation of key aspects on Mesenchymal Stromal Cells (MSCs) isolation and expansion. **(A)** MSC Source Selection; **(B)** Tissue/Cell Collection; **(C)** MSCs Isolation and Expansion.

Given that *in vitro* expansion is always necessary for clinical escalation, we would select an MSC source that ensures large amounts of cells with high proliferation potential and capable of withstanding long periods in culture before acquiring genetic instability or a senescent profile (50). The most common sources of MSCs assessed in clinical trials have been umbilical cord, bone marrow, and adipose tissue (51), and although MSCs isolated from other sources have also being used, there is less experience with them (**Figure 1A**).

### Collection of Starting Material and MSCs Isolation

Friedenstein et al. first described BM-MSCs in 1968 as a population of adherent fibroblast-like cells present in the bone marrow and they are now the most studied source globally (52). Bone marrow harvesting is an invasive procedure that requires local anesthesia with or without superficial sedation, with the iliac crest being the preferred site to obtain larger volumes of BM for clinical applications. The procedure can be performed by multiple punctures and marrow aspirations of small volumes of 1-4 ml with 10-ml syringes prefilled with heparin, or by few or single-site large BM aspiration with needle redirection. Although small repeated aspirations need a longer operation time, in combination with 10-ml syringes they obtain larger MSC yields than BM harvest through single-site puncture aspirated with 50-ml syringes, probably because of blood dilution in the latter (53). Other authors found that the single-stick aspiration method is sufficient to obtain quality marrow aspirates (54). Once obtained, the optimum temperature for maintaining the BM is 2-8°C degrees if overnight storage or shipping is needed (**Figure 1B**).

BM must be processed within 24 hours of collection, although some studies have shown that MSCs derived from cryopreserved marrow have the same growth kinetics and fulfill ISCT criteria as well as fresh marrow–derived MSCs, but further investigation about the effects of cryopreservation on their therapeutic potential is required (55). BM aspirates can be directly cultured, but are more often submitted to a density gradient centrifugation process to isolate BM nucleated cells (BM-NC). Interestingly, both hematocrit and red blood cell release can induce necrosis and apoptosis of MSC (56). In this first step of BM-NC isolation, the yield of cells can vary between different density gradient separation protocols (whether manual or automated). Once obtained, BM-NC must be seeded in a low plating density, about 1-2 x 10^5^ cells/cm^2^, to enhance the proliferation of adherent cell populations at P0. In some cases, positive immunoselection strategies allow the culture of smaller subpopulations of MSCs (**Figure 1C**) (50).

In recent years, both umbilical cord and adipose tissue have gained more ground than BM as MSC sources because of some logistic and functional advantages. UC-MSCs have a higher proliferative and differentiation potential than MSCs obtained from adult tissues and express pluripotency markers that are not present in adult cells (57). UC samples can be stored at 2-8°C and then MSCs can be isolated by explant or enzymatic digestion methods. In the explant method after arteries and vein removal, the remaining tissue and the Wharton’s jelly is cut into small fragments and suspended in culture medium for 7 days in a 37°C humidified incubator with 5% CO_2_. The tissue must be left undisturbed to allow cell migration from the explants while the culture medium must be replaced periodically. For enzymatic digestion, the cord is cut into small fragments and incubated with 500 U/mL collagenase at 37°C in a tissue dissociator. Then, the obtained cells are seeded in culture flasks (58) (**Figures 1B, C**).

Subcutaneous adipose tissue can be easily obtained from donors by in bloc resection (usually discarded as waste in many surgeries) or with a cannula connected to a suction system. In any condition, a 100–500 fold higher number of stem cells compared to BM are yielded (59). Fat removal by liposuction is the preferred harvesting technique for healthy donors and can be combined with ultrasound energy to breakdown adipose tissue facilitate its removal and decrease bleeding and operation time (60, 61). Lipoaspirate must be stored for no longer than 24 h at 2-8°C to maintain the optimal quality of ADSCs (62).

Enzymatic digestion with GMP degree recombinant collagenase followed by centrifugation and washing is the most widespread isolation method for adipose tissue, with a concentration of lyophilized enzyme ranging from 0.075% (w/v) to 0.3% (w/v). This step can be followed by an erythrocyte lysis phase to get rid of erythrocyte contamination. Some protocols improve ADSC isolation and facilitate enzymatic digestion by using mechanical disruption (63), or by replacing enzymatic digestion by mechanical procedures, such as centrifugation, filtration, and micro-fragmentation to minimize costs and to avoid safety issues associated with the use of collagenase (64). ADSCs show more genetic and morphologic stability in long-term cultures and faster proliferation than BM-MSC, even when harvested from the same donor. This is a clear advantage for large scale culture over BM-MSCs in which cultures beyond 20 days and passages beyond 6-7 are associated with senescence (51) (**Figure 1C**).

These tissues and cells used as starting materials for ATMPs may only be obtained in centers authorized by the competent health authority such as collection centers, according to the rules described in section 1.2. All these variables are critical from the beginning of the manufacturing process and each modification must be considered and approved during validations (65) (**Figure 1B**).

### MSC Expansion

The optimal culture conditions for clinical scale production of human MSCs are not standardized across laboratories although it is well known that plate density, culture time and medium composition have a critical influence on the final MSC properties (66), which complicates product comparability among manufacturing centers and extrapolation of results in terms of MSC safety and efficiency across different clinical studies.

Before each culture, the MSCs must have adhered to the culture surface and proliferated, but should not reach over 80% confluence to prevent inhibition by cell-to–cell contact. Accordingly, MSCs have to be plated at a cell density that allows for optimal cellular expansion avoiding continuous premature passages if we plate at high seeding density, or excessively long-term cultures if we plate cell at too low a seeding density. These two situations affect cell proliferation and could lead to senescence or genetic instability (67). Automation of cell cultures for growing large numbers of adherent cells can provide savings in labor costs and improvements in cell quality, a key issue when scaling-up the processes. Bioreactors can enable frequent feeding of the culture, maintaining the levels of metabolites critical for cell expansion under control and allowing a faster and healthier expansion of MSCs than conventional cultures (68–70).

Oxygen concentration is also an important parameter to control. In recent years, hypoxia (3-5%) has been claimed as more physiological environment for cells than normoxia (21%). However, to date, MSCs are mainly cultured under normoxic conditions and reasons to justify the change require validation (71). Alpha- minimum essential medium (α-MEM) or Dulbecco’s modified Eagle’s medium (DMEM) supplemented with fetal bovine serum (FBS) are the gold standard culture mediums for MSC used in most clinical trials. However, xenogenic FBS have some immunological disadvantages and infectious concerns that require controls and validation of each batch. Accordingly, there is interest in the development of serum-substitutes and serum-free media for large scale expansion but taking care to retain MSC characteristics. Cultures of UC-MSCs supplied with 7.5%-10% of activated platelet rich plasma obtained from donor cord blood showed better results than those cultured with standard FBS-containing media. Furthermore, ADSC have been successfully cultured with allogenic platelet lysate generated by freeze-thawing of human platelet concentrates (76–79). However, as the use of hPL has also economic and regulatory concerns, future efforts are directed towards developing standardized GMP-grade formulation with recombinant bioactive molecules to “compensate” for the reduction or lack of serum (76, 77).

## Quality Methods That Ensure Efficiency and Safety

Quality controls ensure the quality of drug products under the rules of the International Council for Harmonization of Technical Requirements for Pharmaceuticals for Human Use (ICH)^2^. Mandatory guidelines for the producers of ATMPs contain important consensuses on the performance of stability studies, the definition of thresholds for impurities testing, and on quality based on Good Manufacturing Practice (GMP) risk management. The quality controls carried out on the ATMPs would be based on the aforementioned guidelines, as well as those dictated by the Pharmacopoeia (78) for performance in a range of tests.

The quality of MSC products is broadly ensured at three different levels: selection of the starting material and raw and packaging materials, control of the manufacturing process (GMP and in-process testing) and the final release testing of the product to ensure patient safety. Selection of the starting material that implies the donation, attainment and testing of human tissues and cells used as starting materials, would be in accordance with the Directive 2004/23/EC (**Table 1**). The ATMPs manufacturer together with the supplier will establish the specifications which in-process controls include: tests performed during the manufacturing process to monitor and, if necessary, adjust the process to ensure that the intermediate/finished product meets its specification. Before the release of MSCs to be administered to patients, quality controls must also be performed to ensure the quality of the final products. Likewise, the excipients used in the manufacturing would be of suitable quality and manufactured under adequate conditions.

MSCs are ATMPs with specific attributes. The first condition to generate a reliable stem cell (MSC) product for clinical trials and routine patient care is to ensure their identity by isolating homogeneous cell populations, following the ISCT recommendations (46). As a living cell product, viability of the MSCs must be ensured in all steps of the manufacturing process and before their administration. The most used test, due to its speed and simplicity of elaboration, is the trypan blue exclusion. Purity is necessary to demonstrate that the cellular population of the drug product does not contain cells other than MSCs (EMEA/CHMP/410869/2006, **Table 2**). Immunophenotyping of the MSCs by flow cytometry according to the ISCT criteria is the most widely used technique.

Potency is a quantitative measure of the biological activity of the product to be tested, which is linked to its biological properties (FDA-2008-D-0520; CPMP/ICH/365/96, **Table 2**). Assessment of these biological properties constitutes another essential step to establish a complete characterization profile of the medicinal product. The biological activity is the capacity of a product to achieve a specific biological effect. Furthermore, the potency test is also the only property that is linked directly to efficacy, shows a correlation with the intended use or predicts the desired therapeutic effect (79). This could be based on *in vitro* co-culture assays to demonstrate the status of MSC activation, and MSC-mediated inhibition of T cell activation or proliferation (80). Unfortunately, it is not clear which is the best potency assay to demonstrate immunomodulatory and regenerative capacities of the MSCs, but this would undoubtedly include tests of safety and stability and, in addition, potency also correlates with the desired effect (FDA-2008-D-050, **Table 2**).

Safety concerns can be derived from the intrinsic characteristics of the ATMPs, the manufacturing process, or the risk of transmitting pathogens to the product.

However, conventional safety studies may not be suitable due to the unpredictable evolution of the cells and/or the *in vivo* behavior of the product; accordingly, both *in vitro* and *in vivo* studies may be required for a safety profile characterization.

On the other hand, the tumorigenic potential of MSCs does not appear to constitute a substantial problem, because short- rather than long-term MSC cultures are used for therapeutic proposes to reduce the duration of *in vitro* MSC expansions (81). In this regard, because most cells can acquire chromosomal aberrations during extensive culture, it would be pertinent to perform a genetic analysis prior to MSC administration. There is a legal requirement to demonstrate the genetic stability of the final cell product. Karyotyping is used to detect abnormal chromosome structure or number. Array-CGH allows a higher resolution in the detection of alterations or copy number changes (82). Indeed, both tests are complementary, because CGH-arrays have a high sensitivity but do not detect polyploidy or balanced translocations, whereas karyotyping detects them but has a lower sensitivity.

Also, safety studies involving microbiological testing (83) must be carried out immediately before packaging or as late as possible during the manufacturing process. In-process testing would also be performed at appropriate steps of the production process such as when changing the storage medium. Microbiological testing includes: testing for aerobic and anaerobic bacteria and fungi (see the *European Pharmacopoeia (Ph. Eur.)*, in particular chapters 2.6.1, 2.6.12, 2.6.13 and 2.6.27); mycoplasma (*Ph. Eur*. chapter 2.6.7) and bacterial endotoxins (according *Ph. Eur*. chapters 2.6.14 and 5.1.10) (78). Although, most of the MSC manufacturing process is open processing, there are also closed manufacturing systems. In all cases without a terminal sterilization process, the environmental microbiological monitoring of cleanrooms is mandatory (EU-GMP-ATMPs, **Table 2**) to minimize risks of microbiological contamination of the product. These monitoring tests include:

-*Volumetric sampling*: Quantifies bacteria and fungi suspended in the air surrounding the open product.
-*Settle plates*: Qualitative evaluation of bacteria and fungi in the air over the plate. At rest and in process conditions.
- *Contact plates*: Qualitative test to detect contamination on the surface of the work area, conducted under uniform pressure for 10 seconds.
*- Swabs*: Qualitative test of the bacteria and fungi on the surface. In this case, the settling plates can be exposed for less than 4 hours during critical operations.
-*Glove prints:* Assessment of the bacteria and fungi contamination of the glove prints (all five fingers) of the operator, after processing or before changing gloves.

Stability testing is required to generate data as well as for establishment of the shelf-life of all the intermediate products subjected to storage and of the finished product. The stability would be demonstrated for the conditions and maximum storage period specified for the MSC product, providing assurance that changes in the identity, purity and potency of the product will be detected (CPMP7ICH7138/95, **Table 2**). Therefore, we use the same test to assess the conditions described above. The intermediate products and cell banks would be tested in a similar way as the finished product. In addition, these quality controls allow for the evaluation of the consistency of batch-to-batch manufacturing.

Manufacturing processes are continuously being improved, especially in the first phases of development of the ATMPs. Depending on the consequences of the changes introduced and the stage of development, comparability studies may be needed to ensure that the changes do not have a negative impact on the product (EMA8CHMP7BWP7534898/2009, **Table 2**). The challenge of these studies is to ensure that the quality, safety and efficacy of the product are not altered by changes in the manufacturing process. The protocol would include molecular characterization, purity, potency and stability assays. A demonstration of comparability does not imply that the quality attributes are identical, but that they are highly similar and any difference between them has no negative impact on the drug product (80). The definition of the strategy for comparability testing must be documented and an experimental plan would be available with written procedures and specifications for each test.

Assessment of the quality of the finished product is mandatory to ensure patient safety. The finished product will not be released for administration until it conforms to the specifications and its quality has been considered satisfactory in accordance with pre-specified requirements. Homogenizing the quality controls carried out on MSCs is critical in order to evaluate their therapeutic efficacy.

## Cell Banks for MSCs

One of the most relevant objectives of cell production units for clinical application is the optimization and improvement of cell culture production yields. Culture conditions can be improved by using different culture media and growth factors, but cell banks can greatly increase the final cell yield (84).

Cell banks allow the storage of intermediate production products that occupy a reduced storage space and that, once thawed, allow a large number of cells to be expanded without the need to resort to primary culture originated from the initial tissue. In addition, if a sequential, or two-tiered, system of cell banks is established, large numbers of cells can be obtained from a small amount of starting material.

In general terms, a Cellular Bank is a collection of approved cell containers, with a uniform composition, which are stored under defined conditions. Each container represents an aliquot of one cell type pool (ICHQ5D, **Table 2**).

According to the EU ATMPs-GMP, cell banks can be classified into:

- *Master Cell Bank* (MCB): a culture of fully characterized cells, which have been obtained from a selected cell population under defined conditions, distributed in containers in a single operation, treated in a way that guarantees uniformity and stored in a way that stability is guaranteed.
- *Working Cell Bank* (WCB): a culture of cells derived from the Master Cell Bank, distributed in containers in a single operation, treated in a way that guarantees uniformity, and stored guaranteeing its stability. Intended for use in the preparation of cell cultures within production processes for clinical and commercial phases.

A good example of the two-tiered system of a *Master Cell Bank* (MCB) and *Working Cell Bank* (WCB) with MSCs is found in Oliver-Vila et al. (85), where the obtained primary culture of 5 x10^6^ Wharton jelly cells derived from one single umbilical cord is described. From this primary culture they could obtain a MCB with 20 aliquots of 2.5 x10^6^ MSCs. One of these aliquots of MCB could be expanded to obtain a WCB with 8 aliquots of 3 x10^6^ cells. Finally, one of the aliquots of WCB could be expanded to obtain 12 doses of 50 x10^6^ cells of final medicine product. With this two-tiered system of cell banks, the authors report a potential culture yield of 96,000 x10^6^ cells from an initial population of 5 x10^6^ Wharton jelly MSCs from one single umbilical cord. Obviously, the success of this bank system is based on the high growth rate of this type of primary culture. Therefore, this approach is the most recommended when MSCs are used in an allogeneic setting and primary cultures have a good growth rate.

Nevertheless, depending on the donor characteristics and the tissue of origin of the primary culture, it may be difficult to obtain such high yields. On the other hand, if the cells are intended for autologous use, it is usually not necessary to obtain large amounts of final product, although if the treatment implies the administration of several doses over time, it may be convenient to generate small cell banks that allow the rapid production of final products without the need to perform new biopsies and primary cultures (86).

For these cases, the EU ATMPs-GMP (**Table 2**) defines the possibility of creating these small cell banks, calling them *Cellular Stocks* (CS). Therefore, CS are those performed by primary cells expanded to a given number of cells to be aliquoted and used as starting material for production of a limited number of batches of a cell-based ATMPs.

### MSCs Cryopreservation, Storage and Traceability

In recent years, considerable experience has been generated worldwide on MSC cryopreservation procedures. Different methods, rates of cooling and compositions of cryoprotectants have been developed (84, 85, 87). The most widely-used cryoprotectant to date is dimethyl sulfoxide (DMSO), although there are different excipient formulations that can give better performances in post-thaw viability (88). 10% DMSO could be supplemented with a buffer containing reagents ranging from 5% Human Albumin, Human Serum or Human Plasma A/B to more complex formulations involving Dextran-40, Lactobionate, Sucrose, Mannitol, Glucose, Adenosine or Glutathione (89). Freezing procedures usually involve controlled rate freezing for optimal cryopreservation.

Another variable to take into account is the container where the cells are cryogenized. The best ones are cryogenization cell bags, but if small volumes must be frozen the standard screw cap cryotube is more common. However, this system is not the most suitable for procedures under GMP, since its closing systems are not safe and can favor pollutant entry (84). Small volume cryopreservation systems are currently being developed in a completely closed system to prevent this from occurring.

The labeling system must ensure traceability of the cryopreserved batch, including the main data that clearly identifies the sample it contains. Labels must be suitable to withstand cryogenic temperatures and must resist erasure due to chemical agents or organic solvents.

Storage for long periods of time requires temperatures below -120°C, usually in the gaseous phase of liquid nitrogen, as the liquid phase can transmit contamination from one cryobag or cryotube to another (84). Nitrogen tanks must be suitable for their function and have clearly differentiated compartments (i.e. racks) to store the different batches without loss or cross-contamination. In addition, a record form must be kept in order to ensure the traceability of the cells, employing a storage inventory system that indicates the exact place where the different aliquots are stored. There must be a qualified storage temperature recording system that activates an alarm when there is a problem with the storage temperature. The cryopreservation unit must have limited access to authorized personnel only.

Once the MSC has been thawed, the final characterization and delivery to the patient must be performed. Post-thawing release criteria should include parameters such as viability, recovery, phenotyping and potency assay (87, 88). In our experience, thawing of cryopreserved cells is a critical step, it must be done quickly. Before their clinical application, cells should be cultured for a passage, although other available protocols also provide optimal therapeutic potential. On the other hand, although some assays have been developed on the basis of the immunomodulatory and anti-inflammatory activity of the secretome generated by apoptotic cells infusing them after thawing (90), in our experience the medium and long-term results are less promising, as the potential generated is limited.

## Small Extracellular Vesicles Derived From MSCs as Cell-Free Therapy

In recent years, the secretome of MSCs, in particular its non-protein fraction consisting of vesicles of different sizes, has attracted attention as a mediator of the paracrine actions of MSCs. Among them, exosomes, also known as small extracellular vesicles (EVs), are nanosized vesicles released by almost all cell types across species (91). MSC-derived EVs (MSC-EVs) are currently being explored as advanced medical products in cell-free therapies for the treatment of acute kidney injury (92), myocardial ischemia (93–95), spinal cord injury (96), hearing loss after noise trauma (97) among other diseases, although few clinical trials are ongoing. MSC-EVs have several advantages over MSCs. For example: i) their smaller size can prevent microvasculature obstruction inherent to the use of MSCs, especially in solid organs. ii) MSC-EVs can cross the blood brain barrier (BBB) extending their use to neurological disorders (98) while MSC cannot (99), iii) although still complex and with a bioactive cargo dependent on the parental sources, they have a significantly simpler composition than MSCs, iv) as non-living biological products, MSC-EVs are more resistant to manipulation than living cells, v) modification of the MSC-EV cargo through the genetic modification of parental cells with associated adeno- or lentivirus vectors exert reduced risk of tumorigenicity after grafting than transplantation of genetically modified cells (100), vi) MSC-EVs can evade phagocytes (101), so reduced doses can be used *in vivo* to achieve a therapeutic response.

### Definition of EVs

The generation of EVs in a reproducible way is not an easy task since multiple parameters ranging from passage number and cell culture conditions to environmental stimuli can induce modifications of their cargo. They also remarked on the relevance of quantitation and single-particle characterization (size, shape and density) by electron microscopy (102) nanoparticle tracking analysis, dynamic light scattering, Z potential quantification (103) and flow cytometry (104), as well as the functional analysis of EVs.

### Large Scale Production of EVs and Control of Heterogeneity

The use of EVs in clinical practice requires the production of large quantities of these biological products, which cannot be achieved with a single donor of parental cells. One strategy can be to use different donors to generate a large batch or to develop strategies to increase EV production. In this context, two different strategies can be adopted. The first one consists of the immortalization of parental cells using hTERT, c-MYC (105) or others. The second approach is based on the modification of parental cells to increase the EV biogenesis and/or potency. There is growing consensus about the need for parental cell modifications to boost EV therapeutic potential. This can be achieved either by modification of the biosynthetic pathway (106) or by stress signals like radiation, oxidative stress or hypoxia, with the latter being the most commonly used (94, 107, 108). Indeed, many investigations have tried to mimic the pathologic environment by conditioning MSCs with pro-inflammatory cocktails (109), low oxygen concentration (110), or HIF1-α overexpression (111, 112). Other strategies, such as the overexpression of miRNAs in parental cells, have also resulted effective (113). Nonetheless, to date, the vast majority of clinical trials used EVs isolated from non-modified MSC primary cultures on a small number of enrolled patients.

With regards to EV isolation, ultracentrifugation is not feasible in a clinical setting, not only because of the difficulty to ultracentrifuge large amounts of EV containing culture media but also because the process induces deposits of soluble proteins that reduce the purity of EV preparations. Size exclusion chromatography or tangential flow filtration techniques can bypass this problem and they are becoming a widely adopted method for EVs isolation in the clinical setting (114, 115).

### MSC-EVs Manufacturing for Clinical Use

As in the case of clinical applications of MSCs, there are still important challenges to be addressed before implementing the use of EVs in a clinical scenario. The main major issues to be solved include: the scale-up of parental cells in sufficient amounts for clinical use, the costs associated with cell culture in GMP conditions, the use of xeno-free culture media and a minimal characterization of these biological products. MISEV14 and updated MISEV18 recommended, as mentioned above, specific criteria for the definition and classification of MSC-EVs. However, they did not provide guidance on the functional testing of their biological activities. In this context, Dr. Gimona’s group provided an extensive list of *in vitro* and *in vivo* potency assays that should be considered before developing clinical trials with a given biological product based on EVs (116). Several factors must be considered during the manufacturing process such as the: i) tissue source, ii) age of donor, iii) passages of parental cells, or if they are primary cultures or have been immortalized, iv) genetic modifications of parental cells, v) priming of parental cell growth factors or culture under hypoxia and vi) isolation procedures of EVs (117). Comparative studies of clinical grade EVs are scarce and the best players together with appropriate strategies to boost MSC-EV therapeutic potential in a clinical setting remain to be elucidated. Therefore, the use of MSC-EVs offers several advantages to MSC administration but, before these biological products can enter into the clinical arena, important obstacles must be resolved from a medicinal product point of view. These include:

1. Control of heterogeneity in EV production by a given parental cell source by defining an optimal range for EV size and composition. This can be achieved by using immortalized parental cell cultures seeded at a given cell concentration with a controlled number of passages and other culture parameters that can influence EV biogenesis.
2. Isolation of MSC-EVs with procedures that minimize protein contaminants including growth factors or lipoproteins that could be co-purified.
3. Preservation of MSC-EV integrity upon scale-up procedures by measuring the degree of aggregation and agglomeration, given that storage conditions including concentration, pH and temperature can induce the fusion of EVs or damage of the lipid bilayer resulting in leakage of the EV cargo.
4. Implementation of GMP procedures to ensure pathogen-free biological products that can be safely used in humans.

In view of the extensive challenges that native or genetically modified EVs need to overcome, novel strategies to design artificial EVs inspired by native biological products are being designed (118). By combining novel drug delivery systems with recombinant surface molecules or synthetic miRNAs, new biological products could be designed. Ideally, artificial nanotechnologies would emulate EVs thus allowing the functional delivery of RNA and other molecules to site-specific targets, since they could be also loaded by integrins and other surface molecules that could guide internalization in host cells and tissues for target-drug delivery (119). These nanotechnologies would recapitulate the favorable characteristics of EVs while reducing heterogeneity and complexity, enabling them to become realistic medical products. Nonetheless, whatever the use of native or synthetic EVs, it is essential to unravel EV structure and composition and to identify relevant molecules for cell-to-cell communication, intracellular uptake and tissue repair and regeneration in order to define the therapeutic product. This will permit the manufacturing processes to be standardized in a move towards the clinical application of these products.

## Transport Systems of ATMPs

Control of the distribution and transport of ATMPs, and specifically of MSCs, is a critical part of the production process for this type of medicament; the process must guarantee the product quality and ensure the conditions are ideal until administration. The main hurdle with these medical products is their condition of being living organisms, which must maintain sterility, viability, proliferation capacity and potential at the moment of patient infusion (83). Thus, not only does the production of these cells imply, as explained previously, the challenge of obtaining a safe and effective product, but possible deficiencies in their transport may also contribute thus generating doubts about the real efficacy of the medicament.

MSC production as an advanced therapy medicament for application in patients must therefore be understood as the whole process from dispatch and reception of the cell source (BM, Adipose tissue, etc.), processing to obtain the active substance and the final product, to the dispatch and reception of the medicament in a hospital setting. All these form part of a larger puzzle and any, even minor, error at any stage could lead to rejection of the medicament batch. In addition, in the case of autologous use, this batch would be unique. Accordingly, the maintenance of transport conditions ensuring medicament quality and safety is a fundamental and necessary step for obtaining good results in the use of these types of medicaments.

It is the producer’s responsibility to define the best conditions for cell stability, including excipient choice and medium, storage temperature and the time these cells are kept in the cited conditions until implantation without losing properties. Distribution of these products is usually carried out by the producer or an outsourced company. In both cases, they must fulfill GDP (GDP, Good Distribution Practices) defined by European Directives, and ISO-21973, specific certification on logistics used in Stem Cell Therapy (**Table 2**). The chosen conditions: excipient, temperature, container type etc., according to GMP rules, are mandatorily in writing, approved and validated.

### Search for the Best Excipients for Conservation and Distribution of MSCs

One of the more critical problems for MSC producers is cell conservation in a suitable medium/excipient from the end of culture to its application in patients. Not only should the medium keep the cells viable with their properties intact, but the form of administration must also be taken into account, since this affects the choice of excipient if a direct infusion is to be performed, which does not require unnecessary manipulation, which could affect sterility. In systemic infusions, the excipient must have very low density, and would ideally be a liquid to avoid complications such as clots. In the case of local cell implantation, the problem is not the density of the excipient but the method of application, namely the caliber or lumen size of the different tools used: catheters/sheaths (measured in French, the equivalent of diameter in mm multiplied by three) or needles (measured in G, size or diameter of the needle). This caliber would be large enough so as not to offer resistance to the product and break the cells by pressure, which would mean the patient receiving only the excipient with dead cells.

In the majority of studies, the most widely used excipients are isotonic solutions included among intravenous solutions administered to maintain electrolyte balance, such as physiological saline, Ringer’s lactate, etc. (14, 15). These media allow the cells to remain stable, sterile, viable, with proliferation capacity and potential until their application; furthermore, they offer easy systemic and local application. As clinical studies with MSCs are moving from a single center to multicenter settings, and even in cases of MSC production with authorization for commercialization, the administration is often performed in clinical centers different to the production centers and, therefore, at some distance away. In these cases, it is essential to maintain optimum product conditions over longer time periods and recently great advances have been made in this area. Currently, some commercial solutions use biopreservative mediums, optimized for conservation and distribution of these products at low temperatures, either in cold (2-8°C) or cryopreserved conditions (-70°C to -196°C). These mediums, which eliminate the need for serums, proteins and cytotoxic products, reduce the product pH at low temperatures, as well as in other conditions, permitting the recovery of ATMPs post-preservation in safe and good quality conditions for their application to patients (120, 121).

### Primary Packaging

As mentioned previously, one of the main properties of MSCs is their capacity to adhere to plastic, which is maintained beyond the production process and represents an important limitation to be considered when choosing the packaging container. Therefore, the chosen containers must be composed of materials with low adherence, certain types of plastic, resin or glass, with a design that allows total and simple recovery of the product, as well as reducing risks of contamination by manipulation. Products have been designed that meet these requirements and also cryopreserve the cells. Some of the most commonly used are: plastic syringes with Luer-Look, specific polymer and resin vials, ethylene vinyl acetate bags (EVA) for lower volumes, etc (120, 122).

### Secondary Packaging

The choice of secondary packaging, must take into account whether the cells are refrigerated or cryopreserved for transportation, and whether the required packaging is multi-use or single use, as the packaging must protect the product but at the same time insulate and be able to maintain the temperature defined as optimal for transport by the cell producer. These types of packaging are usually composed of expanded foam with low thermal conductivity and high resistance to compression, and must be validated either by the cell producer or the distribution company.

Furthermore, the packaging would include a continuous temperature monitoring system during the complete duration of medicament transport, from leaving the Production Unit to its reception by clinical staff responsible for application of the product to patients. For this reason, the delivery must include dataloggers or continuous registries providing essential information on temperature during the delivery, which will be included in the accompanying documentation. This monitoring allows detection of possible variations in temperature, which if serious could affect the product quality.

### Distribution or Transport Flow

GDPs for MSCs in particular and all ATMPs in general, establish mandatory compliance directives aimed at maintaining product quality and safety, so as to implement a rigorous system of quality management by all those involved in cell distribution, thus guaranteeing the quality and integrity of the product (GDPs, **Table 2**).

In the case of obtaining MSCs from different tissues, it is important to clearly establish the distribution flow. In these types of medicaments, a first shipment must be made with an initial container of transport solution for collection of the source tissue (bone marrow, adipose tissue, periodontal ligament, etc.) from the clinical center to the Production Unit. This is followed by a second shipment of the final product (FP) from the Production Unit to the hospital for patient application.

In both cases, the refrigeration units or packaging must be accompanied by the required documentation from the producer. This must include at least one shipment record including the description of the shipment as well as its state, finalized with the reception record by the clinician. It must also include the shipment label with data required by regulators, including the product name, pharmaceutical form, administration method and unit doses; and, finally, a third document with drug release certification and forms for the communication of adverse reactions.

The packaging will also display exterior labels informing on correct positioning of the shipment (upwards arrows); existence (if any) of infectious agents (three half-moons above a circle); if a genetically modified organism (GMO) is shipped it must be accompanied by mandatory, specific labelling, if it is noninfectious biological material (UN3373), as well as including a number indicating that the medicament presents the lowest degree of hazard; labels indicating whether the shipment includes dry ice (UN1845), or a label indicating maximum and minimum temperatures to which the packaging may be exposed in order to maintain adequate conditions in transit and during delays.

## Handling and Delivery of ATMPs for Therapeutic Use

As described in previous sections, since the beginnings of the 21st century a new type of medicine has emerged in which the used products are living cells, a paradigm that has substantially changed both the pharmaceutical industry and clinical practice. On the one hand, the effects of these living medicines occur in the medium to long-term and, on the other hand, their manipulation and application needs important training. In addition, their efficiency is closely linked to the survival of the medical product and, therefore, to their accurate manipulation. For example, the FATT-1 clinical trial for the treatment of complex perianal fistulas with ADSCs failed to obtain statistically significant results owing to incorrect handling and erroneous application of the cells (i.e., use of hydrogen peroxide, vial shaking for cell resuspending, high speed of cell infusion, etc.) by the professionals; errors that do not occur when non-living drugs are tested (123). Remarkably, despite these mistakes, the low percentage of inoculated living cells that survived continued working for at least one year (86).

In this respect, as remarked above, the clinical use of stem cells, mainly MSCs in advanced phases (Phase III, multicenter) of clinical trials, have provided results that do not meet the expectations generated (121, 124). These disappointing results have raised doubts in society about the real capabilities of stem cells. However, further analysis has shown that there are numerous aspects involved, not just the cell product. It is noteworthy that in this type of medicine, a good experimental design is as important as good training in the handling and application of the medicine to fulfill the expectations of success generated by the research laboratories. There are numerous differences between a conventional clinical trial and those using live drugs. Our experience has shown us the enormous difficulty of working with a short life-span product, highly sensitive to external physical factors such as temperature, or to mishandling.

There are many routes to deliver MSCs to patients, but all can be summarized in two general approaches: systemic injection and local injection. Systemic intravenous (IV) injection delivery is the most widely used method because of its few complications. Patients usually receive premedication with intravenous steroids and chlorphenamine, complying with local protocols for the prevention of allergic and nonhemolytic transfusion reactions. If the cells are cryopreserved with DMSO they would be refrigerated and infused as soon as possible after thawing to avoid DMSO toxicity at room temperature, so premedication must be administered and venous access must be ready before thawing. MSCs can be infused through a peripheral vein or a central venous line at a slow infusion rate of around 2-5 ml/min; nevertheless, detailed information on cell handling during intravascular (iv) infusion in published clinical trials is frequently lacking. It is preferred not to use filters or anti-reflux caps in the case of BM-MSCs whereas for IV deliver of ADSCs, 200 micron infusion filters are usually used to retain any clump that might form. Another matter of discussion is the use of subcutaneous reservoirs or long-running plastic based catheters that could lead to some cell retention in the device itself. In this respect, a subsequent flushing with saline solution of the cell bag and the intravascular device is always recommended after cell infusion.

In our experience, the main clinical mistakes detected in the handling of living stem cell products include (i) vigorous shaking of cell vials resulting in cell death by friction, (ii) breach of storage temperature leading to cell senescence or apoptosis, (iii) fast resuspension of the cell pellets resulting in disruption of plasma membranes and cell clumping, (iv) fast injection of cells that also results in cell death due to needle friction, (v) local injection of cells in a hostile environment (i.e., use of hydrogen peroxide as a disinfectant), and (vi) poor location of the cell implant after local delivery that needs critically precise injection. In this respect, the cells must be deposited with precision controlling infusion rate and exact site of delivery, neither too superficial nor too deep. When this step depends on the skill of the surgeon alone, variations occur between centers and clinical trials making standardization and eventually automation of this process essential. In this regard, effective delivery techniques must be considered. It is important to ensure that cell survival after local injection is sufficient to have a therapeutic effect at the site of injury. Therefore, experimental pre-studies for each application are essential (125, 126). Particularly relevant is the culture prior to local injection of MSC cryopreserved with DMSO because the resuspension volume of the cell product in the local injection is insufficient to dilute the toxicity of the cryopreservative; so, it is essential “to refresh” the cells after thawing for safe application. In order to resolve these problems and improve MSC handling we propose the following solutions:

- To have a team of doctors, nurses and supporting staff in charge of cell therapies with training and experience in the handling of live medicines. It is important that the auxiliary staff have been specifically trained in the handling of cells for therapeutic use and are involved in the design and implementation of the logistics for the intervention and administration of the cells.
- Likewise, involvement of the Hospital Pharmacy or the Cell Therapy Area is also important. This department is in charge of receiving the treatment and transporting it to the department where the cells will be administered. These facilities must store the cells properly and dispense them in a timely manner for their correct implantation, including controlled transport.
- The presence of an expert during the first treatments in a center guarantees the proper handling of the cells and their correct administration in each disease and ensures that the process is homogeneously performed from one center to another.
- A subsequent flushing of the intravascular device is recommended after cell infusion. Other requirements include the use of systems with a treated plastic that prevents MSC adherence, as well as glass bottles instead of plastic bags during cell manipulation

## Conclusions and Future Direction

**Figure 2:**
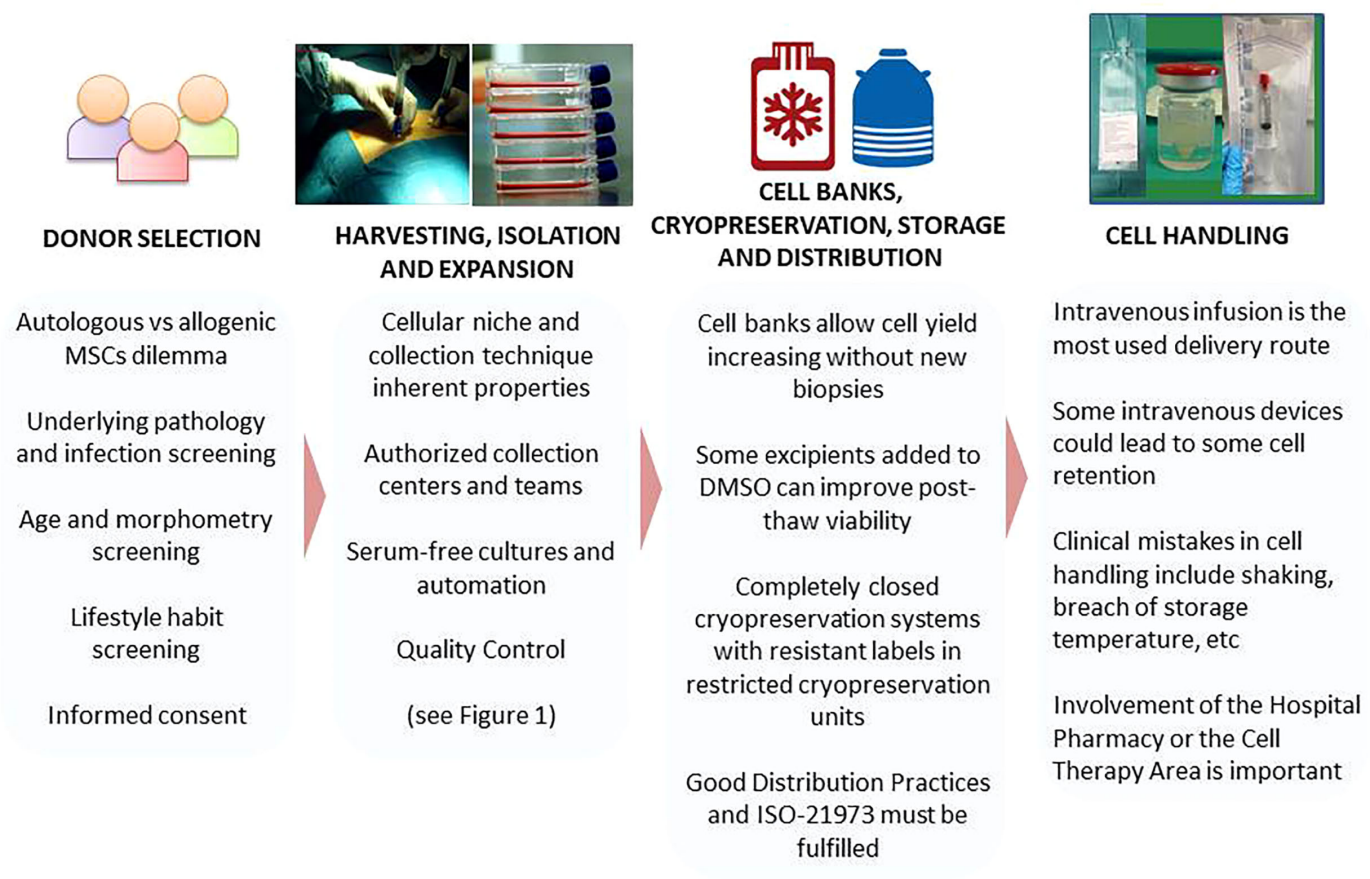
Critical Steps in MSC Manifacturing. (MSCs, Mesenchymal Stromal Cells; DMSO, Dimethyl sulfoxide).

In the present review, we focus on the protocols that allow an adequate manufacturing of MSCs for their application as ATMPs. The flow diagram of **Figure 2** summarizes the main steps of MSC manufacturing. Firstly, we reviewed the regulation for controlling the production, commercialization and application of cellular and gene therapy products, a critical point for ensuring the quality, safety and efficacy of ATMPs. It was particularly important: the definition of ATMPs, the role for donor selection, and the determination of cellular manufacturing under GMP conditions. ATMPs are those containing genes, cells or tissues suffering manipulation and/or cells that may be used in different ways than in the tissues of origin. The aim of donor selection is to guarantee the traceability of donated ATMPs to ensure information is available on the future of the donation. The regulation of cell products generated under GMP conditions is important because they emphasize the significance of a good definition of the products used, from the starting materials, to the collection and shipping of ATMPs. Under the legal and ethical rules dictated by the authorities, we highlight the parameters to define an “optimal donor”, highly dependent on the source of MSCs. For instance, the best adipose tissue-derived MSCs would be isolated from the subcutaneous fat of women under 40 years old, with healthy habits and a BMI lower than 26.8. However, further studies are required to determine the influence of severe and/or chronic diseases on the therapeutic properties of the isolated MSCs, or the age or race of mothers on the quality of UC-derived MSCs. Currently, the MSCs used as starting material to obtain ATMPs can only be isolated in authorized centers, and the processes involved are standardized around the world. On the contrary, the optimal conditions for culturing isolated MSCs are not standardized, constituting a major challenge to improve their therapeutic properties. Cell density in the culture, time of culture and the composition of culture medium are bottlenecks that need critical controls. When many cells are required, this can be controlled by the automation of culture that control metabolites and O_2_ concentration essential for cell expansion. Nevertheless, further studies are required to conclusively determine whether normoxic or hypoxic conditions are the best for MSC cultures, and the culture supplements must be carefully selected, considering that MSC from different sources presumably have distinct needs. In summary, it is important a better definition of the critical quality attributes that reflect in part the known heterogeneity of cultured MSCs. Thus, specific surface markers (i.e., CD200, CD106, CD146, Stro1, CD271), biophysical attributes and genomic markers have been proposed for this evaluation (127).

After MSC production, the quality of ATMPs must be tested according to the available guidelines based on GMPs. The quality of MSC products involves the selection of starting and packaging materials, control of the manufacturing process and testing the quality of the final products. MSC quality is evaluated by measuring purity, potency and safety and is key to the success of the subsequent therapeutic administration. The procedures for achieving these tests are extensively standardized but, especially when cells are long-term cultures, a combination of karyotyping and array-CGH is recommended. In addition, microbiological tests must be performed before packaging and as late as possible during the manufacturing procedure.

Cell banks (i.e., Master Cell Banks, Working Cell Banks and Cellular Stocks) are also necessary for storage of the medicinal products, particularly those to be used in allogeneic conditions. Related to cell banking, numerous procedures have been developed for MSC cryopreservation. Distinct compositions of DMSO supplied with diverse molecules are the most used cryopreservatives. Special containers are used for cryogenization and cell volumes for freezing are a critical feature. In addition, after thawing new challenges arise concerning cell recovery, viability, phenotype and potency. Once again, only authorized personal can manipulate the cryopreserved materials.

Although MSC-EVs have some advantages as therapeutic products over MSCs, their production, maintenance and administration have unresolved challenges. Indeed, a reproducible, standardized generation of MSC-EVs is lacking for several reasons: multiple parameters affect the nature of cargos; MSCs from different donors produce many vesicles and aggregation and fusion of MSC-EVs in large particles is frequent. Moreover, EVs must be produced in large amounts to be used in clinical trials, requiring several donors that result in heterogeneous EVs. Indeed, numerous factors, including MSC source, age of donor, culture conditions and modifications undergone by parental cells can affect the nature of the isolated MSC-EVs. Accordingly, new strategies are required to avoid these problems, by generating “artificial” EVs that maintain the most relevant characteristics of MSC-EVs but reduce their heterogeneity and complexity. The identification of key molecules in MSC-EVs will contribute to define minimal features for improving their therapeutic applications.

In a next future, cell therapy, particularly by the routine administration of MSCs or CAR (chimeric antigen receptor) cells, might become predominant in medicine, but only some centers would be able to produce and supply cells, development procedures to control these shipments critical. Any mistake in this process could result in alterations of the product making its therapeutic application inviable. The selection of excipients, storage and shipping temperature and the container conditions are particularly important. On arrival at hospitals, correct handling of the cells is critical for the success of cellular therapies. Here, we emphasize once again that in this area of medicine the drugs are actually living cells, whose manipulation and administration require special care and training. It is particularly important that the personnel, including auxiliary staff, in charge of the cell therapy and those present during the cell infusion receive special training. As discussed in the text, gentle handling of live products is essential. Emphasis should be placed on the absence of vigorous movements, low infusion rate and, in the case of local injection, optimal choice of injection site based on previous studies. All of this must be achieved with trained personnel in the handling of live medicines. Therefore, it is an unmet need to publish recommendations that standardize a basic protocol for MSC handling worldwide.

## Author Contributions

MF-S, MG-A and AZ made contributions to the coordination and writing of this manuscript. EA, AG-H, ML-P, EV, and PS: manuscript writing. FF-A, DG-O, FP, FS-G, JM and AZ: manuscript review and funding acquisition. All authors accept the published version of the manuscript. All authors contributed to the article and approved the submitted version.

## Funding

This manuscript has been supported by the Instituto de Salud Carlos III (ISCIII) through the project “RD16/0011: Red de Terapia Celular” (Groups: 0001, 0002, 0004, 0005, 0013, 0015, and 0029), from the sub-programme RETICS, integrated in the “Plan Estatal de I+D+I 2013-2016” and co-financed by the European Regional Development Fund (ERDF) “A way to make Europe”, and also by the ISCIII through the project RICORS “RD21/0017;TERAV” (Groups: 001, 002, 003, 006, 009 and 010) that is supported by the Next Generation EU program (Plan de Recuperación, Transformación y Resiliencia); and the Regional Government of Madrid (S2017/BMD-3962, Avancell-CM).

## Conflict of Interest

FS-G has received honoraria and/or research support from Novartis, Kite/Gilead, Celgene/BMS, Pfizer, Takeda and Roche. DG-O is a member of the Advisory Board of Tigenix SAU and received fees from Takeda. ML-P has received honoraria from Novartis, Kite/Gilead and Celgene/BMS. JM has received research support from Roche, Pfizer, Jazz Pharma, Sandoz-Novartis, Gilead, Celgene, and Takeda not related to this manuscript.

The remaining authors declare that the research was conducted in the absence of any commercial or financial relationships that could be construed as a potential conflict of interest.

## Publisher’s Note

All claims expressed in this article are solely those of the authors and do not necessarily represent those of their affiliated organizations, or those of the publisher, the editors and the reviewers. Any product that may be evaluated in this article, or claim that may be made by its manufacturer, is not guaranteed or endorsed by the publisher.

## Footnotes

1
https://www.fda.gov/vaccines-blood-biologics/cellular-gene-therapy-products/approvedcellular-and-gene-therapy-products. (Accessed March 31, 2022).

2
https://www.ich.org/page/quality-guidelines (Accessed March 31, 2022).
